# Conserved Targets to Prevent Emerging Coronaviruses

**DOI:** 10.3390/v14030563

**Published:** 2022-03-09

**Authors:** Fernanda Gonzalez Lomeli, Nicole Elmaraghy, Anthony Castro, Claudia V. Osuna Guerrero, Laura L. Newcomb

**Affiliations:** Biology Department, California State University, San Bernardino, CA 92407, USA; 006041330@coyote.csusb.edu (F.G.L.); 005901892@coyote.csusb.edu (N.E.); 006047752@coyote.csusb.edu (A.C.); 005223695@coyote.csusb.edu (C.V.O.G.)

**Keywords:** COVID-19, coronavirus, zoonosis, antiviral

## Abstract

Novel coronaviruses emerged as zoonotic outbreaks in humans in 2003 (SARS), 2012 (MERS), and notably in 2019 (SARS2), which resulted in the COVID-19 pandemic, causing worldwide health and economic disaster. Vaccines provide the best protection against disease but cannot be developed and engineered quickly enough to prevent emerging viruses, zoonotic outbreaks, and pandemics. Antivirals are the best first line of therapeutic defense against novel emerging viruses. Coronaviruses are plus sense, single stranded, RNA genome viruses that undergo frequent genetic mutation and recombination, allowing for the emergence of novel coronavirus strains and variants. The molecular life cycle of the coronavirus family offers many conserved activities to be exploited as targets for antivirals. Here, we review the molecular life cycle of coronaviruses and consider antiviral therapies, approved and under development, that target the conserved activities of coronaviruses. To identify additional targets to inhibit emerging coronaviruses, we carried out in silico sequence and structure analysis of coronavirus proteins isolated from bat and human hosts. We highlight conserved and accessible viral protein domains and residues as possible targets for the development of viral inhibitors. Devising multiple antiviral therapies that target conserved viral features to be used in combination is the best first line of therapeutic defense to prevent emerging viruses from developing into outbreaks and pandemics.

## 1. Introduction

Coronaviruses are ubiquitous in bats, and spillover events into humans occurred in 2003 with SARS, again in 2012 with MERS, and most recently in 2019 with SARS-CoV-2. The initial coronavirus outbreaks were warnings for the COVID-19 pandemic, which to date has claimed over 5.97 million lives worldwide and over 954,500 in the United States (as of 2 March 2022) [1]. Indeed, we may see SARS-CoV-2 become endemic and require yearly vaccination updates, much like human influenza [2]. Similar to the influenza virus, given the nature of coronaviruses, novel human coronaviruses are likely to emerge again in the future.

Yearly vaccination provides excellent protection against targeted influenza A and B circulating strains, but there are years where the circulating influenza viruses change too much, and the vaccine protection loses efficacy. As reported by the CDC, influenza vaccine efficacy over the past decade ranged from a high of 60% to a low of 19% [3]. The mRNA COVID-19 vaccines demonstrated excellent vaccine efficacy, with 63% protection from infection and 94% protection from severe disease after two doses when first introduced [4]. However, as SARS-CoV-2 continues to circulate, the virus continues to change. Indeed, sera tested from people even after the two-dose mRNA COVID-19 vaccine regime did not maintain the antibodies necessary to neutralize all of the circulating SARS-CoV-2 variants. In particular, P.1 (gamma) and B.1.351 (beta) exhibited little neutralization from vaccine-induced humoral immunity [5]. Variant B.1.617.2 (delta) also escapes vaccine-induced neutralizing antibodies [6], lowering the vaccine’s overall efficacy. Indeed, a third dose of mRNA COVID-19 vaccine was found to be required to protect against these new variants, including B.1.617.2 (delta) [7], and the most recent variant of concern, B.1.1.529 (omicron) [8]. Thankfully, in addition to antibody-mediated immunity, vaccines also induce cell-mediated immunity, important in CoV-2 protection, where T-cells recognize infected cells [9]. Therefore, the three doses of mRNA COVID-19 vaccines provide a robust overall immune response to protect against severe disease caused by all of the SARS-CoV-2 variants that have so far emerged, with better than 90% protection against hospitalization [7].

As demonstrated by the decreased vaccine efficacy as new variants emerge, vaccines may not protect as well against emerging novel coronaviruses. While next generation vaccine platforms, including mRNA vaccines, will improve the response time against emerging viruses, vaccine production may still take months to develop, manufacture to scale, and administer to populations during an emerging outbreak. Further, while vaccines are safe and effective, vaccine hesitancy is a major threat to world health [10], and ensures susceptible individuals remain in the population. Lastly, vaccines do not help individuals already infected.

The first therapeutic line of defense against emerging viruses is antiviral medications. Antivirals are essential to treat infected individuals, especially in the absence of a vaccine. Our current arsenal of antivirals did little to limit the destruction caused by SARS-CoV-2, emphasizing the need to develop robust antiviral therapies to employ during novel viral outbreaks. While the pandemic has enhanced research into coronavirus antivirals, much work remains to prepare for the next coronavirus outbreak. Antivirals that target the conserved activities or interactions of viral molecules are likely to provide efficacy against multiple variants and strains, especially when employed in combination. The identification of unique antiviral targets conserved among coronaviruses is the first step to prevent future pandemics caused by emerging coronaviruses. Here, we review the coronavirus molecular life cycle, consider approved and promising antiviral inhibitors, and identify novel conserved antiviral targets.

## 2. Materials and Methods

To identify valuable protein targets within coronaviruses, we performed polymorphism analysis of essential viral proteins to define the most conserved residues across human and bat β-coronaviruses. Using the sequence variation analysis tool from the National Institute of Allergy and Infectious Disease-sponsored Virus Pathogen Database and Analysis Resource [11], ViPR, we analyzed the amino acid sequence of seven essential coronavirus proteins to identify conserved residues. The parameters were only selected for complete genomes from bat and human hosts, starting from the year 2000 for each protein name. The retrieved sequences for each protein were analyzed and returned with a polymorphic score assigned for every amino acid position. Amino acid residues with low polymorphic scores have high conservation and were identified as residues of interest.

Using Mol* Viewer [12], we then located the conserved residues within the crystal structure obtained from the Protein Databank (PDB) [13,14]. Mol* Viewer is a tool integrated into the PDB that allows for the three-dimensional visualization of the conserved residues in protein crystal structures. We highlighted the conserved residues that are also accessible on the protein structure. Conserved residues of interest were analyzed with the ASAview algorithm tool integrated into the PDB viewing software. The ASAview tool uses the DSSP program [15] to accurately predict the distribution of residues and solvent exposure in the protein, also known as the amino acid Accessible Surface Area (ASA) [16]. The accessibility cutoff for residues was arbitrarily selected depending on the relative abundance and accessibility of the conserved residues, which varied within each protein. Some proteins show greater quantities of highly conserved residues and we highlight here the most conserved and accessible residues in each protein. Each protein with the highlighted residues were downloaded as a PNG file and a complete figure with labeling designed through BioRender (BioRender, Toronto, ON, Canada).

## 3. Results

### 3.1. Conserved Antiviral Targets in Coronavirus Molecular Life Cycle

#### 3.1.1. Inhibition of Viral Entry

**Figure 1:**
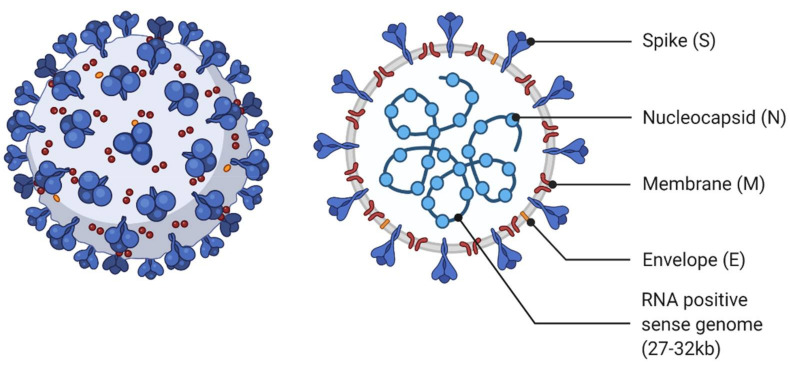
Coronavirus virion. The coronavirus virion contains the long (27–30 kb) viral single-stranded positive sense RNA genome encapsulated by the viral nucleocapsid (N) protein. The RNA/nucleocapsid complex is protected by the viral envelope comprised of viral envelope (E), membrane (M), and spike (S) proteins. The S protein includes the receptor binding domain (RBD) to target host cells. Created with BioRender.

Coronaviruses are enveloped viruses, with one positive-sense, single-stranded RNA genome segment ranging from 27 to 32 kb. The genome is bound by viral nucleocapsid phosphoprotein (N), which is surrounded by the viral envelope, composed of viral envelope protein (E), membrane protein (M), and the spike glycoprotein (S) (Figure 1). The envelope spike glycoprotein is responsible for the entry of the coronavirus into the host cell. The spike protein functions as a trimer. Each spike protein can be separated into two domains: S1 and S2. S1 contains the receptor binding domain (RBD), which interacts with the host cell surface receptor. The RBD is variable among the coronavirus family. SARS-CoV-2 S1 RBD recognizes ACE2 and likely DPP4 [17]. ACE2 is mainly found in the heart, lung, kidney, and alveolar epithelial type II cells, which explains the effect of SARS-CoV-2 on respiratory function. S2 contains the fusion peptide (FP) and two heptapeptide repeat sequences (HR1 and HR2); in contrast to the RBD, the FP domains are highly conserved among the coronavirus family.

**Figure 2:**
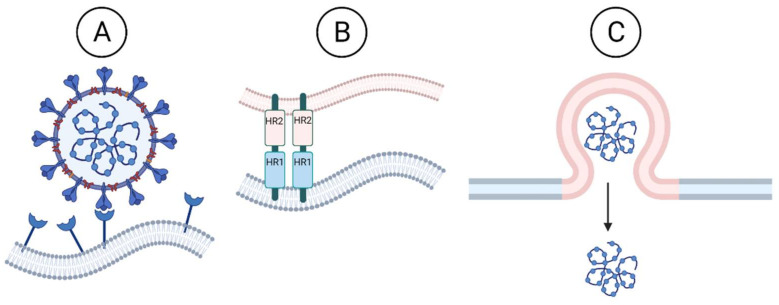
Coronavirus entry. To release the viral RNA genome, the coronavirus must bind to the target host cell, fuse the viral and host membranes, and release the viral genome into the host cytoplasm. (**A**) Coronavirus S protein receptor binding domain (RBD) binds with the host receptor on cell surface. SARS-CoV-2 S protein binds host ACE2. (**B**) S protein binding with host receptor exposes the S protein heptad repeat (HR) domains for fusion. HR1 and HR2 domains pull the cell and viral membranes together to fuse. (**C**) Fusion results in release of viral genome into the host cell cytoplasm. Created with BioRender.

The interaction between the host receptor and the RBD of the spike allows host cell proteases to cleave the spike into the S1 and S2 subunits. Some coronavirus spike proteins contain an additional furin cleavage site to facilitate host cleavage and viral entry into host cells. The SARS-CoV-2 spike protein contains a furin cleavage site, unlike SARS-CoV, that is not required for infectivity, but contributes to SARS-CoV-2 increased transmission [18]. Once S1 and S2 are cleaved, the FP in S2 is exposed. This exposure prompts a conformational change where the fusion peptide inserts itself into the host membrane. At this point, HR1 is close to the host cell membrane and HR2 is close to the viral membrane. HR1 and HR2 then bind together to make a six-helix structure at the fusion area. Afterwards, HR2 pulls itself back into HR1 so that the viral membrane is being pulled into the host cell membrane, and they fuse. This fusion event releases the viral RNA genome into the host cytoplasm (Figure 2).

The spike protein binds to the host cell surface receptor through the receptor binding domain (RBD). The RBD of the spike protein is variable among different coronavirus strains, which, expectedly, use different host receptors [19]. Yet, this step is successfully blocked with specific antivirals targeting this interaction. One approach, exemplified by dalbavancin, a lipoglycopeptide antibiotic, was shown to block SARS-CoV-2 virus entry in mice and rhesus macaque models by binding and blocking the host ACE2 receptor [20]. However, the ACE2 receptor has an endogenous host function, so more commonly the virus is targeted with neutralizing antibodies that bind the viral spike protein through the RBD and tag the virus for clearance by the immune system. Neutralizing antibodies can be isolated from patient serum or engineered and purified in the laboratory. Regeneron Pharmaceuticals and Lilly Pharmaceuticals both engineer neutralizing antibody cocktails given emergency use authorization to combat SARS-CoV-2 infection [21,22]. These antivirals are specific to the coronavirus strain, or even the variant, making the RBD unsuitable to target new, emerging coronavirus strains and variants. To exemplify this, the omicron variant contains 36 amino acid changes to spike [23], limiting the efficacy of these treatments for omicron infection. Indeed, the US-FDA has limited the use of monoclonal antibodies to patients not infected with omicron, because the treatment has limited efficacy against the changed spike protein RBD of omicron [24].

The development of pan-CoV fusion inhibitors is an active research area and important goal to stave off emerging coronavirus outbreaks [25]. The S2 protein domain is highly conserved within the coronavirus family, and both the fusion peptide (FP) domain and heptad repeat sequences (HR1 and HR2) are excellent targets for antiviral therapies against emerging coronaviruses [26] (Figure 2). For example, the HR1 region of the human immunodeficiency virus-1 (HIV) Gp41 fusion protein is targeted by Fuzeon^TM^, a fusion inhibitor approved by the FDA for clinical use against HIV-1. Another class of antivirals, called anchor inhibitors, target the conserved FP domain of Gp41 and are under development [27]. Overall, the inhibition of the fusion step is a possible target that should be further studied.

#### 3.1.2. Inhibition of Viral Proteases

**Figure 3:**
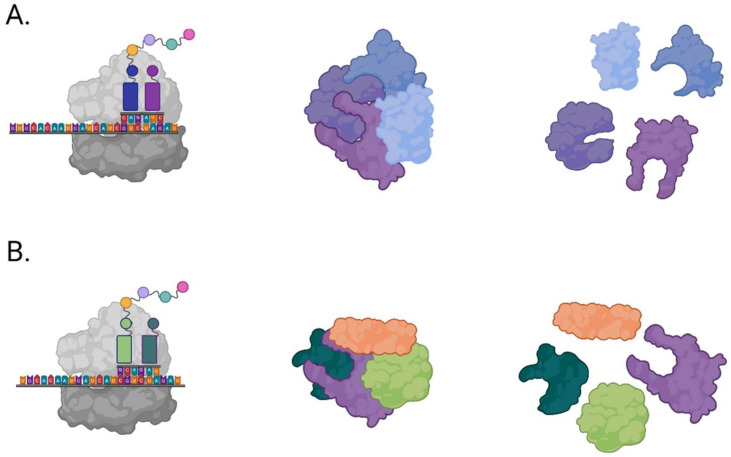
Coronavirus initial translation and polyprotein cleavage. Upon the release of the coronavirus RNA genome into the host cytoplasm, host ribosomes initiate the translation of viral proteins. (**A**) Translation of the viral genome results in expression of viral polyprotein pp1a, which encodes essential viral proteases that cleave the viral polypeptides into individual functional proteins. (**B**) Programmed translation frameshift results in expression of viral polyprotein pp1ab, which is cleaved by the expressed essential viral proteases into individual functional proteins. Note: figure is an illustration, not an accurate depiction of coding sequence or protein structure. Created with BioRender.

Translation occurs first as the coronavirus RNA genome is modified with a 5′ cap and 3′ poly A tail, similar to eukaryotic mRNA. Upon the release of the RNA genome, the viral RNA is immediately recognized by the host ribosome and translated into a large polyprotein, pp1a. Viral proteases are among the first to be translated within pp1a and serve to cleave the large polypeptide into functional proteins [28]. During translation, a programmed ribosomal frameshift sometimes occurs, resulting in the translation of a second large polyprotein, pp1ab [29]. The viral proteases cleave these two large polyproteins, pp1a and pp1ab, into the additional necessary viral nonstructural proteins (Figure 3).

Protease inhibitors are successful antiviral therapies for other viruses that rely on polyprotein cleavage as a mechanism of viral protein expression, such as Olysio^TM^ for the hepatitis C virus. There are numerous FDA-approved protease inhibitors to target HIV-1 protease activity. Unfortunately, these protease inhibitors did not demonstrate clinical efficacy against SARS-CoV-2 [30]. However, the crystal structure of the main protease of SARS-CoV-2, a 3C-like protease, termed Mpro or 3CLpro and encoded by nsp5, was rapidly solved in attempts to identify protease inhibitors [31]. Optimized CoV protease inhibitors showed efficacy in vitro and in mice against several human coronaviruses [32]. Indeed, Pfizer has asked and received FDA emergency approval of an optimized coronavirus protease inhibitor, Paxlovid [33]. Protease inhibitors show real promise as potential therapy against emerging coronaviruses. 

**Figure 4:**
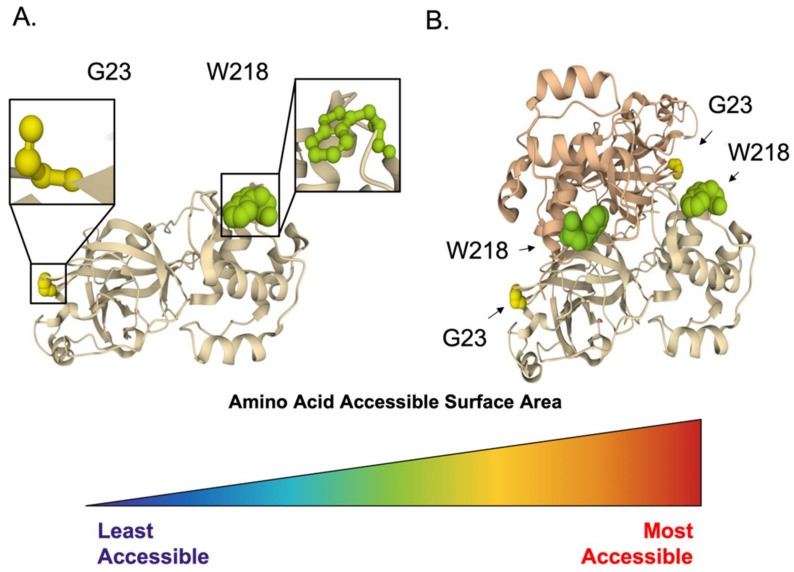
Conserved and accessible amino acids of CoV Protease. Identified conserved residues of interest within coronavirus Nsp5 protease crystal structure (PDB ID: 5GWY) shown as a monomer (**A**) and a dimer (**B**). Two conserved and accessible residues, G23 and W218, are highlighted with molecular surface representation and colored by amino acid accessible surface area (ASA) ranging from the least accessible (dark blue) to the most accessible (red).

In addition to the protease activity of nsp5, two highly conserved and accessible residues were identified from our polymorphism and structure analysis (Figure 4). Amino acid sequences for Nsp5 in bat or human hosts deposited between January 2000 and July 2021 were obtained using ViPR, with the search name “nsp5”. The database search resulted in 405 retrieved sequences. Amino acid sequence polymorphism analysis revealed 15 highly conserved residues within Nsp5, with a polymorphic score of 0. Of those 15, a total of 12 residues were resolved in the crystal structure (PDB ID: 5GWY) [34]. Residues with an accessible surface area of less than 50 Å^2^ were omitted to highlight the two most exposed amino acids. We found both G23 and W213 highly conserved, with a polymorphic score of 0. G23 is somewhat accessible with an ASA of 53 Å^2^ for both the monomer (Figure 4A) and dimer (Figure 4B), but W213 was even more accessible with an ASA of 96 Å^2^ and 105 Å^2^ within the dimer (Figure 4B). We propose that these residues may be involved in essential Nsp5 interactions and may provide an additional target within Nsp5 to inhibit viral replication.

#### 3.1.3. Inhibition of Transcription and Replication 

**Figure 5:**
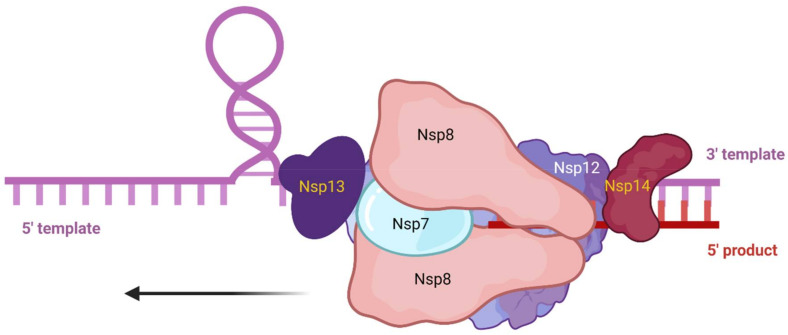
Coronavirus replicase. Coronavirus replicase complex includes the polymerizing subunit, Nsp12, with two copies of Nsp8 and one copy of Nsp7 to complete the functioning RNA-dependent RNA polymerase. Nsp13 interacts with Nsp12 and contains helicase activity to relieve RNA secondary structure in the template RNA. RNA synthesis occurs 5′ to 3′ as indicated by the arrow. Nsp14 is a novel exonuclease to “proofread” the RNA products and methyltransferase activity involved in methylation of the viral mRNA 5′ cap structure. Note: figure is an illustration, not an accurate depiction of the replicase protein structure. Created with BioRender.

Coronavirus genome is replicated and transcribed by the RNA-dependent RNA polymerase, encoded by nsp12, along with additional nonstructural proteins (Figure 5). Nsp12 is comprised of the conserved RdRP at its C-terminus, with characterized palm, fingers, and thumb domains, along with two additional domains, an N-terminal nucleotidyltransferase (NiRAN) domain and an interface domain [35]. These N-terminal domains are not well characterized. Based on sequence homology, the NiRAN domain is speculated to contain a kinase fold, and may possess kinase or phosphotransferase activity, which could be targeted to inhibit viral replication [36]. Although the N-terminal domains of Nsp12 are not well characterized, the viral RNA replication and transcription mechanisms of the coronavirus replication machinery are defined. The active site for RNA polymerization is located on the palm domain within the C-terminus of Nsp12, and is formed by five conserved Nsp12 elements known as motifs A–E. Motif C binds to the RNA 3’ end and contains the residues D760 and D761, which are required for RNA synthesis [35].

Nsp12 alone is not sufficient for viral RNA synthesis, and the coronavirus RdRP holoenzyme includes additional non-structural proteins, including two copies of Nsp8 and one copy of Nsp7. The two copies of Nsp8 interact differently with the RNA template and with Nsp7 to improve the processivity of Nsp12. Amino acid substitutions within Nsp8 and Nsp7 that disrupt interaction with Nsp12 demonstrate the importance of these additional nonstructural proteins for replicase function. Three residues within SARS-CoV Nsp8—K58, P138, and R190—are essential for the interaction with Nsp12 and crucial for SARS-CoV genome replication [37]. Additional evidence of the importance of Nsp7 and Nsp8 for coronavirus RdRP processivity comes from SARS-CoV, where experiments with pairwise combinations of Nsp7 or Nsp8 with Nsp12 displayed no significant polymerase activity; expression of all three proteins was essential [37].

**Figure 6:**
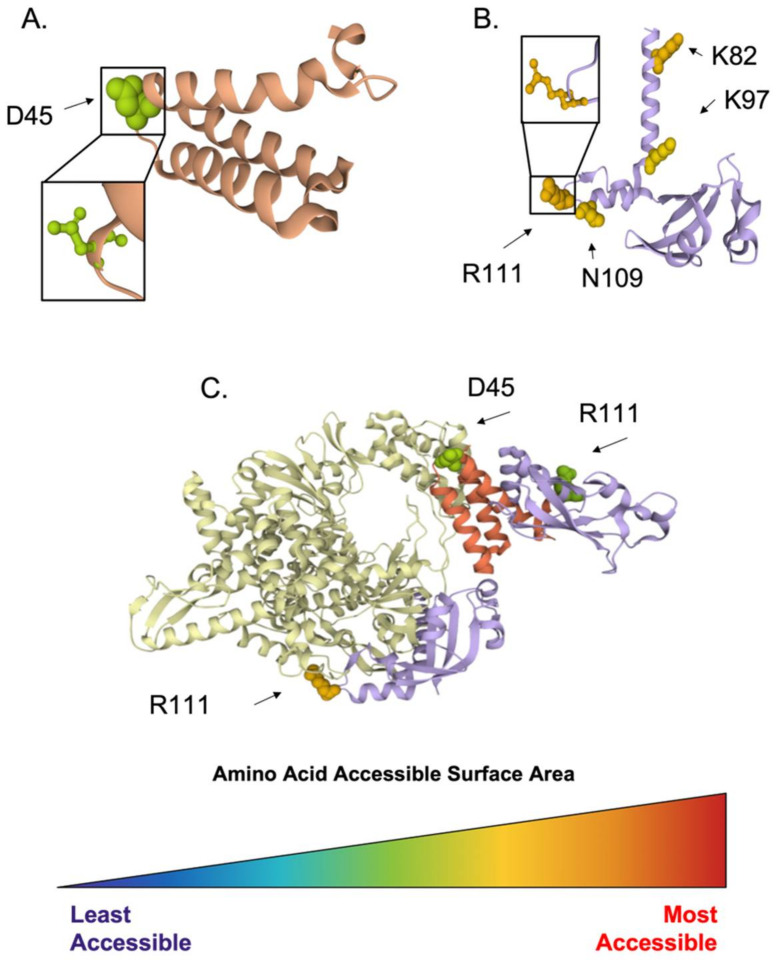
Conserved and accessible amino acids of CoV replicase. Identified conserved and accessible residues of interest within coronavirus Nsp7 and Nsp8 highlighted with molecular surface representation and colored by amino acid accessible surface area (ASA), ranging from the least accessible (dark blue) to the most accessible (red) in their respective monomer crystal structures (**A**,**B**) and in complex with the viral RNA-dependent RNA polymerase (Nsp12) (**C**). (**A**) Nsp7 (PDB ID: 6NUR) with the most conserved and exposed amino acid highlighted, D45. (**B**) Four highly conserved and exposed residues within the Nsp8 structure are highlighted, K82, K97, N109, and R111, with magnification of the most accessible residue, R111. (**C**) The most conserved and accessible residue of Nsp7 (red-orange) and Nsp8 (purple) highlighted in the replicase holoenzyme (PDB ID: 6NUR), complexed with Nsp12 (white).

While essential for the functioning RdRP holoenzyme, both of these small nonstructural proteins reveal a few highly conserved and accessible residues (Figure 6). A search for Nsp7 amino acid sequences isolated from bats or human hosts deposited between January 2000 and December 2020 was performed on ViPR, using the protein name “nsp7”. The search resulted in 405 retrieved sequences, which were analyzed for polymorphism. Throughout the Nsp7 sequence, five residues were determined to be highly conserved, with polymorphic scores of zero. The crystal structure of the viral RdRP holoenzyme, PDB ID: 6NUR [38], comprises the viral RdRP Nsp12 along with Nsp7 and two copies of Nsp8. Four of the nsp7’s identified amino acids were resolved in the crystal structure; however, three were excluded due to an ASA of 0 Å^2^, leaving the most conserved and accessible residue, D45, highlighted (Figure 6A,C).

Using identical host and date parameters to those used with nsp7, but changing the gene name to search for “nsp8”, retrieved 7911 sequences. Polymorphism analysis identified 57 highly conserved amino acids comprising Nsp8, with a polymorphic score ranging from zero to two. Two amino acids reported as essential for Nsp8 interaction with Nsp12 [37] were identified as highly conserved in our analysis, with a polymorphic score of 0, namely, K58 and R190. All 57 conserved residues were resolved on the crystal structure; however, many residues were internal. Due to the number of conserved residues, we arbitrarily omitted residues with an ASA less than 110 Å^2^, resulting in just four highly conserved and accessible residues to highlight: K82, K97, N109, and R111 (Figure 6B,C).

**Figure 7:**
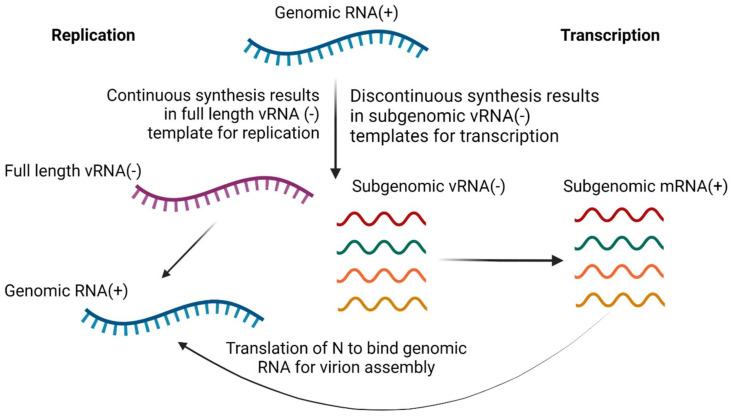
Coronavirus transcription and replication. The coronavirus replicase employs discontinuous synthesis to make a set of subgenomic vRNAs of negative sense from the viral positive-sense RNA genome. The subgenomic vRNAs serve as a template for transcription of subgenomic viral mRNAs. The replicase also synthesizes continuous full-length negative-sense vRNAs, which serve as a template for replication of genomic RNAs. Created with BioRender.

The CoV RdRP holoenzyme is assisted by the activities of additional nonstructural proteins, Nsp13 and Nsp14 (Figure 5). Nsp13 is an RNA helicase that interacts directly with Nsp12 to increase processivity by eliminating the RNA template secondary structure. Nsp14 contains a novel exonuclease activity to remove mismatches and improve fidelity during replication of the very lengthy coronavirus genome [37]. Nsp13 and Nsp14 also have activities involved in processing the 5′ cap for viral mRNA transcripts [39,40]. The transcription of CoV mRNAs occurs in a discontinuous manner. First, the plus strand genome is used to make a set of subgenomic negative sense RNAs that contain the same 5′ leader sequence and serve as a template for transcription of viral mRNAs by the viral RdRP complex (Figure 7). The viral plus-sense subgenomic mRNAs are modified with a 5′ cap, likely due to the RNA 5′-triphosphatase activity of Nsp13 [40] and methyltransferase activity of Nsp14 [39], along with a 3′ polyA tail, synthesized from a long stretch of Us on the 5′ end of the viral RNA template and elongated by the adenyltransferase activity of Nsp8 [41]. The robust template switching and polymerase jumping during discontinuous transcription is one explanation for the observation that coronaviruses frequently recombine [42].

The coronavirus replicase complex is a highly conserved and essential target for antivirals. The conserved active site within CoV RdRP is targeted by nucleotide analogs such as remdesivir and ribavirin [43]. However, due to the exonuclease activity of Nsp14, these analogs do not have the same efficacy against coronaviruses compared with the efficacy against other viral RdRP. Coronaviruses have the largest known RNA genomes, and to improve fidelity during viral replication, coronaviruses evolved a 3′ to 5′ exonuclease, encoded by nsp14, to provide “proofreading” during viral genome replication. Nsp14 recognizes incorporated chain-terminating nucleotide analogs as a mismatch, resulting in the 3′ to 5′ exonuclease removal of the incorporated analog, making this antiviral strategy less effective against coronaviruses. However, the viral exonuclease itself is a potential antiviral target, and when inhibited, will increase the efficacy of nucleotide analogs [43], an example of the impact of combinatorial therapy.

Fortunately, another nucleotide analog, the active form of molnupiravir, is incorporated into the growing RNA, but unlike the active forms of remdesivir and ribavirin, the incorporation is not chain-terminating. Molnupiravir is converted in the body to the active nucleotide analog, NHC-5’ triphosphate. This analog can base-pair with purines G and A and is incorporated into the new RNAs in place of cytosine or uracil, escaping correction by Nsp14 [44]. Subsequent use of this RNA as a template will incorporate either G or A, as both can base-pair with the template nucleotide analog, resulting in too many errors to support the viral life cycle. Molnupiravir is administered orally and can be given to patients early on in infection to reduce illness and hospitalizations [45]. Molnupiravir was developed to counter the influenza virus, but has been shown to exhibit antiviral activity against coronavirus and is likely effective against additional RNA virus families because this drug targets a conserved and essential function of viral RNA-dependent RNA polymerases. As expected, molnupiravir proved effective against SARS-CoV-2 variants of concern in a hamster model [46] and against other human coronaviruses, including SARS and MERS in mice models [47], and seasonal coronaviruses, HCoV-NL63, HCoV-OC43, and HCoV-229E in cell culture [48]. Merck sought and gained FDA emergency use approval after very promising clinical trials [49,50,51]. Molnupiravir is an important antiviral to prevent and control emerging coronaviruses and other emerging RNA viruses.

**Figure 8:**
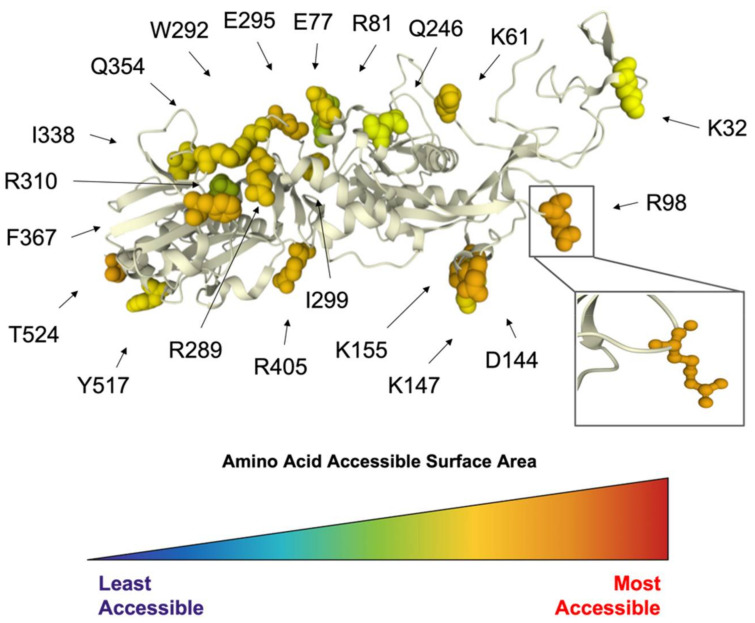
Conserved and accessible amino acids of CoV exonuclease. Identified conserved and accessible residues within coronavirus exonuclease highlighted with molecular surface representation and colored by amino acid ASA ranging from the least accessible (dark blue) to the most accessible (red) in the Nsp14 crystal structure (PDB ID: 5C8U). Twenty of the most accessible residues are highlighted, with R98 being the most surface-exposed.

Nsp14 is unique to the nidovirus family, of which coronavirus is a member, and serves to “proofread” the viral RNA products during viral RNA synthesis. Our polymorphism analysis reveals an entire conserved surface of Nsp14, along with additional accessible residues (Figure 8). A search of the ViPR database for Nsp14 sequences from bat or human hosts deposited between January 2000 and March 2020 using the protein name “exonuclease” yielded 7827 sequences, which were then analyzed for sequence polymorphism. Nsp14 is highly conserved and a total of 248 residues showed a polymorphic score of either zero or one in our analysis. All conserved residues were resolved in the crystal structure PDB ID: 5C8U [52]. To limit residues of interest, amino acids with an accessible surface area less than 100 Å^2^ were excluded, leaving 20 highly conserved and accessible residues highlighted. Nsp14 displays multiple conserved and accessible areas for further study as a potential antiviral target (Figure 8).

The coronavirus helicase, encoded by Nsp13, is another essential and highly conserved component of CoV replicase machinery under consideration to target with small molecule inhibitors [53]. Nsp13 is intriguing as a general CoV target, because Nsp13 is a multi-functional protein with a zinc-binding domain (ZBD) within the N-terminus and a helicase domain with conserved motifs of superfamily-1 (SF1) helicases in the C-terminus. Both the NTPase and RNA helicase activities of SARS-CoV-2 Nsp13 are effectively inhibited by bismuth salts, supporting Nsp13 activities as viable antiviral targets for further study [54]. The N-terminus of Nsp13 forms a Zn^2+^ binding cluster conserved in all coronaviruses and required for functional interaction with Nsp8 [55]. 

**Figure 9:**
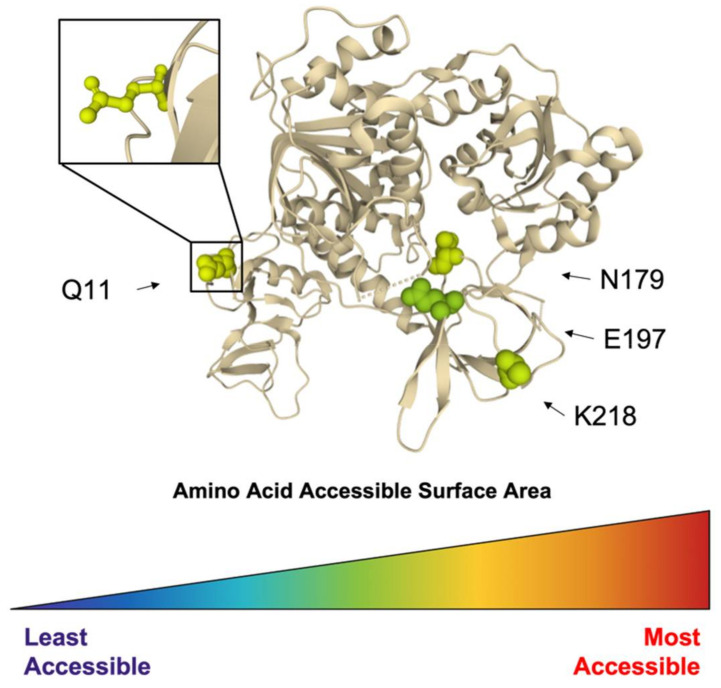
Conserved and accessible amino acids of CoV helicase. Identified conserved and accessible residues within coronavirus helicase highlighted with molecular surface representation and colored by amino acid ASA, ranging from the least accessible (dark blue) to the most accessible (red) in the Nsp13 crystal structure (PDB ID: 6ZSL). Four highly conserved residues are highlighted within the monomer by accessibility—Q11, N179, E197, and K21—with Q11 having the greatest ASA.

In addition to essential helicase function, Nsp13 polymorphism analysis revealed four highly conserved residues, Q11, N179, E197, and K218, accessible for interactions and/or modifications (Figure 9). Helicase amino acid sequences isolated from bats or humans, deposited to ViPR between January 2000 and December 2020, were retrieved using the search name “nsp13”. This search brought back 404 sequences, which were subsequently analyzed for amino acid polymorphism. A total of 48 residues within Nsp13 returned with a polymorphic score of zero, with many residing internally within the protein. All of the residues are resolved in the crystal structure PDB ID: 6ZSL [56]. After analyzing the most conserved residues, only those with the greatest exposure are represented in Figure 9. All four highlighted residues presented an ASA greater than 80 Å^2^, with the most exposed, Q11, with an ASA of 101 Å^2^. These conserved and accessible residues may represent interaction domains essential for viral replicase function and should be considered targets for novel antivirals.

The nucleocapsid phosphoprotein (N) binds full-length genomic RNA and plays a role in the regulation of viral transcription and replication. The phosphorylation of the viral N protein by host glycogen synthase kinase-3 (GSK-3) is required for the production of longer subgenomic mRNA transcription and full-length RNA genome replication. Phosphorylated N recruits host RNA helicase DDX1 to facilitate readthrough for the synthesis of longer subgenomic mRNA and genomic RNA [57]. As enough N protein is synthesized and phosphorylated, the switch from transcription to genome replication occurs. During genome replication, the viral RdRP synthesizes a full-length negative-sense copy of the positive-sense genomic RNA. This serves as a template for the robust synthesis of genomic positive-sense RNA genomes that are rapidly bound by N proteins in preparation of virion assembly and exit (Figure 7). Coronavirus nucleocapsid phosphoprotein is an essential protein for viral genome replication and packaging genomes into new virions.

#### 3.1.4. Inhibition of Virion Assembly and Exit

**Figure 10:**
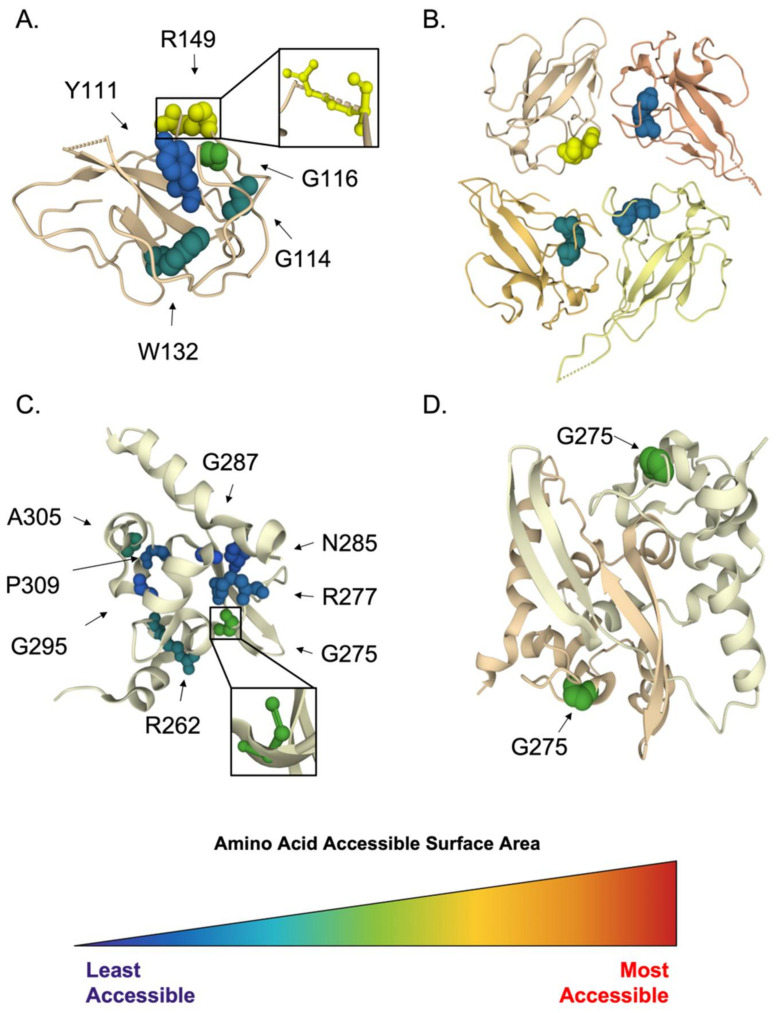
Conserved and accessible amino acids of CoV nucleocapsid phosphoprotein. Identified conserved and accessible residues within coronavirus nucleocapsid phosphoprotein are highlighted with molecular surface representation and colored by amino acid ASA, ranging from the least accessible (dark blue) to the most accessible (red) within the SARS-CoV-2 N crystal structure. (**A**) Monomer of the N-terminus of N (PDB ID: 6WKP). Most residues are deep blue or dark green, indicating that they are inaccessible and internal to the protein structure. Arg149 is the most accessible and magnified here. (**B**) Multimer of the N-terminus of NP with Arg149 highlighted and color coded by accessibility. In the multimer, at least one subunit retains Arg149 accessibility. (**C**) Monomer of the C-terminus of N (PDB ID: 6ZCO) with eight conserved residues highlighted by accessibility. Most residues are blue and dark green, buried within the protein, and less accessible, while G275 is the most accessible and magnified here. (**D**) Dimer of the C-terminus of N with the most accessible residue, G275, highlighted in green. Both residues in their respective monomer structures display similar accessibility when in the dimerized form.

The exit pathway for all β-coronaviruses begins with newly synthesized viral genomic RNA coated with viral N proteins during replication to form a helical ribonucleoprotein. Nucleocapsid phosphoprotein amino acid sequences isolated from bats or human hosts and deposited between January 2000 and December 2019 were retrieved from ViPR using the protein name “nucleo”. This search retrieved 669 sequences, which were analyzed for polymorphism. Fifteen amino acid residues in the N-terminus were identified as highly conserved, with a polymorphic score of zero. Comparison to the crystal structure, PDB ID: 6WKP [58], shows that 13 of these were resolved, mostly internal to the protein and likely playing a role in protein folding and tertiary structure (Figure 10A). However, residue Arg149 is accessible, with an ASA of 129 Å^2^ in the monomer (Figure 10A), and likely involved in RNA binding when pictured in the multimer structure (Figure 10B). Indeed, this residue was identified as one of the multiple basic amino acids essential for N-RNA interaction [59]. The inhibition of N-RNA interaction would inhibit both viral replication and virion assembly. We propose this highly conserved residue be considered a target for disrupting the essential N-RNA interaction.

**Figure 11:**
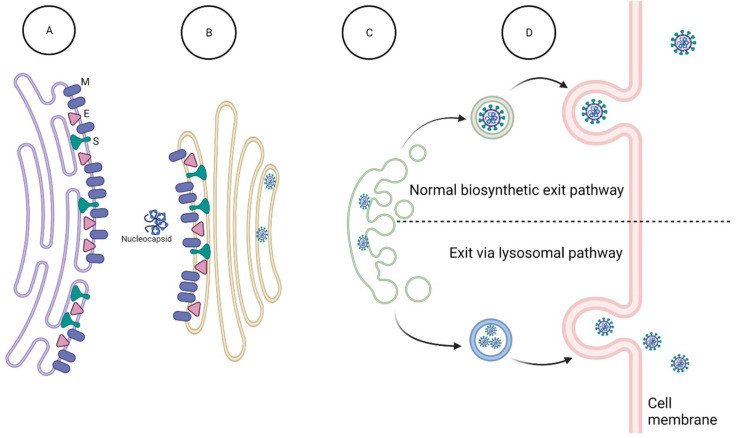
Coronavirus assembly and exit. Translation results in coronavirus protein expression followed by viral genome replication. (**A**) Upon translation, N binds viral genomic RNA to form the nucleocapsid. Translation of M, E, and S proteins occurs in the endoplasmic reticulum (ER). (**B**) Viral assembly is initiated by viral protein–protein interactions between M and N of the nucleocapsid, along with M interaction with both E and S in the ER Golgi intermediate complex (ERGIC). (**C**). Virions traffic to the Golgi apparatus for glycosylation and post-translational modifications of viral surface proteins. (**D**). Virions are either released in vesicles as part of the normal cellular secretory exit pathway, or virions are released via the cellular lysosomal pathway. Created with BioRender.

The ribonucleoprotein is next assembled with other structural proteins to form the new virion. The viral envelope (E), membrane (M), and spike (S) proteins are translated by ribosomes in the endoplasmic reticulum (ER) and travel to the ER Golgi intermediate compartment (ERGIC). When in the ER/ERGIC, virus proteins traffic to the Golgi apparatus and trans-Golgi network (TGN) for glycosylation and other post-translational modifications [60,61]. The M protein is the main organizer behind virion assembly. M maintains essential interactions with itself, N, E, and S, to lead virion assembly [60,62,63]. M interacts with N bound to RNA as part of the helical ribonucleoprotein to stabilize and package the genome into the newly assembling virions (Figure 11).

The N protein sequence polymorphic analysis revealed eight amino acid residues as highly conserved in the C-terminus of N, with a polymorphic score of three or less. When compared to the crystal structure, PDB ID: 6ZCO [64], most of the conserved residues are internal to the protein and likely play a role in protein folding and tertiary structure (Figure 10C). However, Gly 275 is somewhat accessible even within the dimer structure, with an ASA of 28 Å^2^ (Figure 10D). We hypothesize this nonpolar residue may be important for N-hydrophobic interaction with other viral proteins, and as such, should be explored further as a potential target to inhibit viral replication.

M interactions are essential for virion assembly and the inhibition of these interactions inhibits virion assembly, serving as antiviral targets [62]. M interaction with S ensures the spike stays in the ERGIC for incorporation into new virions. The interactions between M and E are essential in assembly, and the expression of M and E alone are sufficient to make viral-like particles (VLP). Conversely, mutations that disrupt M and E interaction do not allow for VLP formation [65]. Additional interactions required to assemble new virions are under investigation as targets for novel antivirals, such as the interaction between S and N [66]. The coronavirus conserved protein residues required for interactions essential for new virion assembly are rational targets for novel antivirals to inhibit virus production.

**Figure 12:**
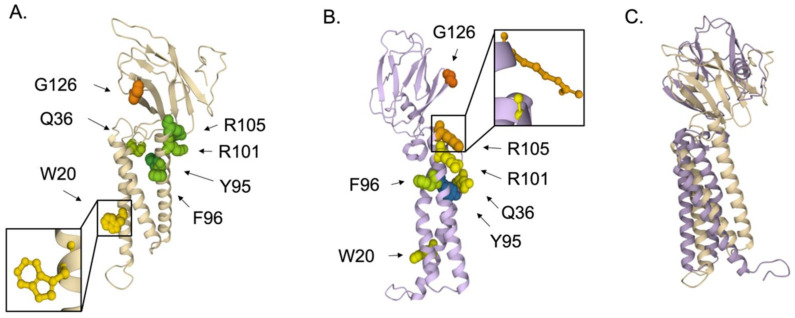
Conserved and accessible amino acids of CoV membrane protein. Identified conserved and accessible residues within the coronavirus membrane protein highlighted with molecular surface representation and colored by amino acid ASA, ranging from the least accessible (dark blue) to the most accessible (red) within the SARS-CoV-2 M crystal structure. (**A**) M structure modelled through I-TASSER (PBD ID: QHD43419). Seven residues are highlighted, with the most surface-exposed residue, W20, magnified. (**B**) M structure modelled through tr-Rosetta (PDB: TBSD A 1861983 SM5109). The same seven amino acids were identified and are highlighted. (**C**) Superimposed structures of M derived through I-TASSER (tan) and trRosetta (purple).

We identified seven highly conserved residues in the M protein of coronaviruses, with W20 the most accessible (Figure 12). Membrane protein amino acid sequences from bat or human hosts deposited between January 2000 and December 2020 were obtained using ViPR, with the search name “M”. This search retrieved 9356 sequences, which were then analyzed for sequence polymorphism. The analysis identified 33 highly conserved residues with a polymorphism score of one, two, or three. While a crystal structure for the CoV membrane was not identified in the Protein Data Bank, we found computationally solved structures from Zhang Lab generated using I-TASSER modeling software [67]. Structure QHD43419 predicts the placement of all the residues. However, the amino acids with an ASA less than 70 Å^2^ were excluded to focus on the seven most exposed residues (Figure 12A). We compared the I-TASSER model with the computationally solved structure using trRosetta [68,69]. We propose W20, with an ASA of 157 Å^2^, and G126, which is highly accessible but with an ASA of 72.96 Å^2^ as it is a smaller amino acid, are likely important in virion assembly interactions and could be targeted by small molecule inhibitors. W20 is within a transmembrane domain and may be involved in M dimerization [70]. Small-molecule and anti-TMD peptides can inhibit interactions within membranes [71], and this approach to inhibit M dimerization would block virion assembly. G126 is within the C-terminal domain (CTD) of M, a flexible region of the protein [70] that we speculate could serve to interact with other proteins during the viral life cycle.

After virion assembly in the Golgi apparatus/TGN, it is assumed that coronaviruses use vesicles of the biosynthetic secretory pathway to track to the plasma membrane and egress [72], similar to other enveloped RNA viruses, such as the hepatitis C virus, dengue virus, and West Nile virus [73]. However, evidence suggests that rather than the biosynthetic secretory pathway, at least some coronaviruses use a lysosomal, Arl8b-dependent exocytic pathway for release into the extracellular environment. GRP78/BIP, an ER chaperone that facilitates coronavirus infectivity, is co-released with β-coronaviruses through this pathway [74]. These newly assembled virions are shuttled to the lysosomes, deactivate lysosome function, and exit via lysosome trafficking [74] (Figure 11). Lysosomal trafficking may be exploited as a target of novel antivirals. For example, lysosomal alkylating small molecules inhibit the cytopathic effect caused by SARS-CoV-2 in cell lines. The small molecule VPS34-IN-1, an inhibitor of class III phosphotidylinositide 3-kinase vacuolar protein sorting 34 (VPS34) identified in yeast, blocks the maturation of endosomes. VPS34-IN-1 potently inhibited the SARS-CoV-2 cytopathic effect at concentrations that were not cytotoxic. Additionally, bafilomycin A1, the classic inhibitor of V-ATPase that prevents acidification of the lysosome, is highly cytotoxic, but nevertheless blocked the SARS-CoV-2 cytopathic effect at nontoxic nanomolar concentrations. Combined, the data suggest that endocytosis and the maturation of endosomes toward the lysosome are critical routes for viral exit and can be targeted without cytotoxic effects [75]. In addition to the conserved viral protein activities and interactions as antiviral targets, cellular secretion pathways hijacked by coronaviruses may be inhibited to block viral spread. 

## 4. Discussion

Viruses evolve rapidly, and emerging viruses are a threat to human health and the global economy, as illuminated by the current global COVID-19 pandemic caused by SARS-CoV-2. Similar to the influenza virus, recognized as a constant emerging threat since the 1918 pandemic, coronaviruses have multiple animal hosts. Although the molecular mechanisms vary, both viruses contain RNA genomes that change quickly during replication, including frequent mutation, which can result in antigenic drift and either reassortment of the influenza RNA genome segments or recombination of the large single RNA genome of coronaviruses, leading to antigenic shift. Immune pressures also influence antigenic evolution, and although influenza and coronaviruses have different molecular mechanisms of antigenic drift and shift, both viruses undergo similar rapid rates of antigenic evolution [76]. These molecular mechanisms result in these viruses jumping from animal species into humans on a regular basis; with coronavirus, SARS jumped from bat to civet to humans in 2003 [77], while MERS jumped from bat to camel to humans in 2012 [78]. Although the intermediary animal remains unconfirmed for SARS-CoV-2, a bat origin is highly likely, and emergence directly from bats is probable [79]. Given the ease with which these viruses undergo rapid change, some of which result in zoonosis, it is also likely these viruses will emerge with new strains and subtypes that threaten human health and the economy in the future. 

Antivirals are the first line of therapeutic defense against a novel emerging virus, but antiviral resistance is a concern. For example, the first class of antivirals against influenza, the M2 ion channel inhibitors, are no longer efficacious. According to the Centers for Disease Control, 100% of H3N2 influenza viruses circulating in 2009–2010 and 99.8% of 2009 pandemic H1N1 influenza viruses were resistant to M2 inhibitors. Even the second class of influenza antivirals, neuraminidase inhibitors, have already documented resistant isolates [80,81]. These isolates are also resistant to the M2 ion channel inhibitors, as might be expected given the selection of resistance to the M2 ion channel inhibitors occurred first. Thankfully, a third class of influenza antiviral, endonuclease inhibitors, is approved to combat emerging human influenza viruses [82,83].

To tackle the issue of resistance to antivirals, many viruses are countered with combinatorial therapies that simultaneously target multiple proteins or activities required for viral replication. This strategy is less likely to allow the emergence of a triple resistant isolate than selection with one antiviral pressure at a time. For example, HIV has developed resistance to individual antiviral treatments, but therapeutic strategies that use a combination of three or more antiretroviral (ARV) drugs from at least two different HIV drug classes, for example, targeting the protease, integrase, and replicase at the same time, have proved effective [84]. Success with combinatorial antiviral therapies is also seen with direct-acting antivirals against HCV, which combine inhibitors to the viral polymerase, the viral protease, and a third viral nonstructural protein [85]. Indeed, deaths from either HCV or HIV are in decline [86,87], in part due to the available combinatorial therapies. These victories demonstrate that using combinatorial therapies is the best way to combat rapidly changing RNA viruses, including both influenza viruses and coronaviruses. To combat emerging coronaviruses, more viral targets need to be identified, characterized, and screened against potential inhibitors as the basis for the development of novel antivirals that can be used in combination to prevent emerging and re-emerging coronaviruses. This need is recognized in the scientific community, as highlighted in a recent Nature news report [88].

The molecular life cycle of coronaviruses present opportunities to develop novel inhibitors to block essential and conserved viral molecular activities and interactions. Conservation indicates importance. Conserved activities and interactions across the coronavirus family are high-priority targets to stop emerging coronaviruses, as conserved targets are less likely to tolerate change that leads to resistance. We examined several coronavirus proteins because it is imperative to discover multiple targets and employ combinatorial antiviral therapies to prevent emerging coronaviruses. 

Nsp5 and the viral replicase are targeted by the approved antiviral therapies Paxlovid and Molnupiravir, respectively. Antiviral resistance has yet to emerge, but studies examining the safety of combinatorial therapy in these two antivirals may demonstrate this approach to be more effective and less likely to result in the emergence of resistant viruses. Nsp13, encoding the viral helicase, is a conserved target being actively pursued [53]. While a promising drug target, RNA helicases also exist in the host cell, and therefore the cytotoxicity of inhibitors is a heightened concern. Nsp14, on the other hand, is unique to coronaviruses and may serve as a more practical drug target [89]. The M protein interactions also represent promising antiviral targets [65]. While all proteins examined here should be studied further to develop an arsenal of antiviral therapeutic targets, we propose the CoV N protein should be considered a high priority antiviral target for the development of novel antivirals. N is important in both replication and assembly, participating in essential interactions with multiple viral proteins. The inhibition of these interactions with a combination of small-molecule inhibitors could provide a combinatorial therapy targeted to the different domains and interactions of one viral protein, N, essential in multiple steps of the viral life cycle. N is being actively pursued as a CoV antiviral target [90].

Here, we reviewed the molecular life cycle of coronaviruses, discussed the current state of antiviral therapies to target SARS-CoV-2, and identified additional conserved and accessible residues of essential viral proteins that should be further studied to serve as targets for the development of novel antivirals. Targeting multiple activities and interactions conserved among bat and human coronaviruses with antivirals will provide protection against even emerging coronaviruses, leading to better global outcomes during the inevitable next coronavirus emergence.

## Data Availability

The datasets used and analyzed during the current study are available from the corresponding author in Excel format upon reasonable request.
